# Post-COVID-19 condition 3 months after hospitalisation with SARS-CoV-2 in South Africa: a prospective cohort study

**DOI:** 10.1016/S2214-109X(22)00286-8

**Published:** 2022-08-09

**Authors:** Murray Dryden, Caroline Mudara, Caroline Vika, Lucille Blumberg, Natalie Mayet, Cheryl Cohen, Stefano Tempia, Arifa Parker, Jeremy Nel, Rubeshan Perumal, Michelle J Groome, Francesca Conradie, Norbert Ndjeka, Louise Sigfrid, Laura Merson, Waasila Jassat

**Affiliations:** a Division of Public Health Surveillance and Response, National Institute for Communicable Diseases, Division of the National Health Laboratory Services, Johannesburg, South Africa; b Centre for Respiratory Diseases and Meningitis, National Institute for Communicable Diseases, Division of the National Health Laboratory Services, Johannesburg, South Africa; c Right to Care, Centurion, South Africa; d School of Public Health, Faculty of Health Sciences, University of the Witwatersrand, Johannesburg, South Africa; e School of Pathology, Faculty of Health Sciences, University of the Witwatersrand, Johannesburg, South Africa; f Clinical HIV Research Unit, Faculty of Health Sciences, University of the Witwatersrand, Johannesburg, South Africa; g Department of Medicine, Division of Infectious Diseases, Faculty of Health Sciences, University of the Witwatersrand, Johannesburg, South Africa; h Divisions of General Medicine and Infectious Diseases, Faculty of Medicine and Health Sciences, Stellenbosch University and Tygerberg Hospital, Cape Town, South Africa; i South African Medical Research Council—CAPRISA HIV–Tuberculosis Pathogenesis and Treatment Research Unit, Centre for the AIDS Programme of Research in South Africa, University of KwaZulu-Natal, Durban, South Africa; j Drug-Resistant Tuberculosis, Tuberculosis & HIV Directorate, National Department of Health, Pretoria, South Africa and University of KwaZulu-Natal, Durban, South Africa; k Global Support Centre, International Severe Acute Respiratory and emerging Infections Consortium, Nuffield Department of Medicine, University of Oxford, Oxford, UK; l Pandemic Sciences Centre, International Severe Acute Respiratory and emerging Infections Consortium, Nuffield Department of Medicine, University of Oxford, Oxford, UK

## Abstract

**Background:**

Post COVID-19 condition (PCC), as defined by WHO, refers to a wide range of new, returning, or ongoing health problems in people who have had COVID-19, and it represents a rapidly emerging public health priority. We aimed to establish how this developing condition has affected patients in South Africa and which population groups are at risk.

**Methods:**

In this prospective cohort study, we used the DATCOV national hospital surveillance system to identify participants aged 18 years or older who had been hospitalised with laboratory-confirmed SARS-CoV-2 infection in South Africa. Participants underwent telephone follow-up assessment at 1 month and 3 months after hospital discharge. Participants were assessed using a standardised questionnaire for the evaluation of symptoms, functional status, health-related quality of life, and occupational status. We used negative binomial regression models to determine factors associated with PCC.

**Findings:**

Of 241 159 COVID-19 admissions reported to DATCOV between Dec 1, 2020, and Aug 23, 2021, 8309 were randomly selected for enrolment. Of the 3094 patients that we were able to contact, 2410 (77·9%) consented to participate in the study at 1 month after discharge. Of these, 1873 (77·7%) were followed up at 3 months after hospital discharge. Participants had a median age of 52 years (IQR 41–62) and 960 (51·3%) were women. At 3 months of follow-up, 1249 (66·7%) of 1873 participants reported new or persistent COVID-19-related symptoms, compared with 1978 (82·1%) of 2410 at 1 month after hospital discharge. The most common symptoms reported at 3 months were fatigue (50·3%), shortness of breath (23·4%), confusion or lack of concentration (17·5%), headaches (13·8%), and problems seeing or blurred vision (10·1%). On multivariable analysis, the factors associated with persistent symptoms after acute COVID-19 were being female (adjusted incident rate ratio 1·20, 95% CI 1·04–1·38) and admission to an intensive care unit (1·17, 1·01–1·37).

**Interpretation:**

Most participants in this cohort of individuals previously hospitalised with COVID-19 reported persistent symptoms 3 months after hospital discharge and a significant impact of PCC on their functional and occupational status. The large burden of PCC symptoms identified in this study emphasises the need for a national health strategy. This should include the development of clinical guidelines and training of health-care workers for identifying, assessing, and caring for patients affected by PCC; establishment of multidisciplinary health services; and provision of information and support to people who have PCC.

**Funding:**

Bill & Melinda Gates Foundation, UK Foreign, Commonwealth & Development Office, and Wellcome.

## Introduction

Although there is a growing understanding of COVID-19 and risk factors for severe disease and death, less is known about ongoing and long-term complications. There has been debate about the most appropriate nomenclature and clinical criteria for the long-term complications of COVID-19, with various terminology used over time, including long COVID, long haulers, and post-acute sequelae of COVID-19. WHO has done a global Delphi study to arrive at a consensus on the name and clinical definition of this COVID-19-related condition. Post-COVID-19 condition (PCC) “occurs in individuals with a history of probable or confirmed SARS-CoV-2 infection, usually 3 months from the onset of COVID-19 with symptoms that last for at least 2 months and cannot be explained by an alternative diagnosis”.^1^ WHO also clarified that the symptoms might be a new onset, after initial recovery from an acute COVID-19 episode, or persist from the initial illness, and that they might fluctuate or relapse over time.

Cases of persistent symptoms after acute COVID-19 were described as early as May, 2020,^2^ and our understanding of the condition continues to improve through research. Initial estimates suggested that one in ten people infected with SARS-CoV-2 will have new or persistent symptoms beyond 4 weeks of their acute illness, irrespective of the initial disease severity.^3^ A 2021 systematic review,^4^ which included 57 studies, reported pulmonary sequelae, neurological disorders, mental health disorders, functional mobility impairments, and general and constitutional symptoms. About half of patients had PCC for more than 6 months.^5^ The most prevalent sequelae included impaired concentration, generalised anxiety disorder, fatigue, and muscle weakness and functional mobility disorder. Another study found that at 7 months post-COVID-19 onset, 45% of patients had not returned to previous levels of work and continued to have significant symptom burden.^6^

**Research in context**

**Evidence before this study**

We searched PubMed for any research on the long-term consequences of COVID-19 from Jan 1, 2020, to Dec 1, 2021. Search terms were “Post Acute Sequelae of SARS-Cov-2” OR “PASC” AND “COVID-19” OR “SARS-Cov-2” OR “Post Acute Sequelae of COVID” OR “COVID-19 Sequelae” OR “Long Haul COVID” OR “COVID Long Haul*” OR “Long COVID” OR “Long Term COVID” OR “Chronic COVID Syndrome” OR “Post COVID Syndrome” OR “Post COVID-19 Neurological Syndrome” OR “Post-Acute COVID-19 Syndrome”. We did not find any studies related to the long-term consequences of COVID-19 in South Africa at the time of submission. Additionally, the underlying pathophysiology, prognosis, and duration of persistent COVID-19-related symptoms were not understood and no universal clinical definition had been recognised. As a result, it became increasingly apparent that urgent research, particularly within the African context, was necessary to help design appropriate strategies aimed at providing a clinical framework to assist those affected in lower-income settings.

**Added value of this study**

To our knowledge, this is the largest prospective, observational cohort study of individuals admitted to hospital with COVID-19 that aimed to describe the long-term consequences of COVID-19 in the African context. We found that the number of patients with at least one post-COVID-19 symptom after hospital discharge decreased from 82% at 1 month to 67% at 3 months after hospital discharge. The three most commonly reported symptoms 3 months after hospital discharge were fatigue, shortness of breath, and confusion or lack of concentration. Being female was associated with all five post-COVID-19 condition (PCC) outcomes examined, namely new or persistent symptoms, persistent breathlessness, self-reported non-recovery, new or worsening disability, and anxiety or depression. Other factors associated with persistent symptoms included intensive care unit admission. These data are shared with the International Severe Acute Respiratory and emerging Infections Consortium, along with 18 other countries. This allows for multinational cooperation on data sharing and analysis that can contribute to global understanding of the long-term consequences of COVID-19.

**Implications of all the available evidence**

These early findings show that, although some had achieved a full recovery with no persistent morbidity, about two-thirds of patients who were admitted to hospital for COVID-19 in South Africa still had significant subjective morbidity 3 months after hospital discharge. Ongoing clinical follow-up with these patients at 6 months and 12 months will provide more data on the prevalence of PCC, and future analyses will include comparison of PCC among patients who did not require hospitalisation (mild COVID-19).

The frequency, clinical picture, and effect of PCC could vary in different settings as a result of different patient genetic and social backgrounds. The effect of PCC could be worse in Africa because people often have unreliable income and poor access to health services. The burden of PCC in South Africa could be substantial, given that by July 27, 2021, 2 391 223 SARS-CoV-2 cases had been reported,^7^ while serology studies suggest that more than 70% of people had a SARS-CoV-2 infection at the end of the third wave (May to September, 2021).^8^ However, to our knowledge, no published studies have described the epidemiology of PCC in South Africa, and few studies from Africa on the subject have been published. As part of a multicountry study coordinated by the International Severe Acute Respiratory and emerging Infections Consortium (ISARIC), we established a prospective cohort of participants with SARS-CoV-2 infection for serial follow-up after hospitalisation to determine the prevalence of and risk factors for PCC among individuals hospitalised with laboratory-confirmed SARS-CoV-2 infection in South Africa.

## Methods

### Study design and population

This was a prospective, observational cohort study using an ISARIC open-access tool that was locally adapted to follow up participants with COVID-19 in South Africa.^9^

We used the DATCOV national hospital surveillance system, developed by the National Institute for Communicable Diseases (NICD) and the National Department of Health, to identify participants admitted to hospital who had SARS-CoV-2 infection and to obtain baseline patient data on demographic characteristics, comorbidities, and hospital admissions. The study population included individuals who had a positive RT-PCR assay or rapid antigen test for SARS-CoV-2, were admitted to hospital for a minimum of 1 day in the public or private health sectors from all provinces of South Africa, and were discharged between Dec 1, 2020, and July 27, 2021. We did not routinely do viral sequencing to establish infecting SARS-CoV-2 variants; however, on the basis of South African sequencing data, the two most common variants during the study period were the beta variant (B.1.351; epidemiological week 1 to 20) and delta variant (B.1.617.2; epidemiological week 21 to 30).^10^ Ethnicity was determined by self-identification and categorised in line with the Statistics South Africa classification, including Black, White, Mixed, Indian, and other.

Participants were eligible for inclusion irrespective of reason for hospital admission, including those admitted due to COVID-19 symptoms or with incidental SARS-CoV-2 infection identified during the hospitalisation. Of all eligible, discharged, adult participants with contact details available, a random selection of participants was invited by telephone for participation in this study. Random sampling was done with a computer-generated list of eligible participants. Participants aged 18 years or older who consented verbally to participate in the study were included. The study was approved by the University of the Witwatersrand Human Research Ethics Committee (HREC M201150). Approvals were obtained from all provinces through the National Health Research Database.

### Measurements instruments

We used a standardised case report form (CRF) and follow-up protocol developed by ISARIC in collaboration with members of the global long COVID support group for demographic variables, comorbidities, COVID-19 vaccination status, symptoms during hospitalisation, current health status, acute COVID-19 complications, new or persistent symptoms, and lifestyle and socioeconomic variables. The CRF was piloted with patients in three different countries before it was finalised. The CRF contained validated tools to establish quality of life (measured by EQ-5D-5L), dyspnoea (assessed using modified Medical Research Council dyspnoea scale)^11^ and difficulties in functioning (using the UN–Washington Disability Score).^12^ We used the Washington Group Short Set on Functioning questions^12^ to measure changes in short-term disability (seeing, hearing, walking, remembering, communication, and self-care).

### Data collection

Participants were initially enrolled by means of a telephone assessment at 1 month after hospital discharge, with additional scheduled follow-up assessments at 3, 6, and 12 months post-hospital discharge. Verbal consent was obtained and recorded and, where possible, interviews were done in the language of the participants’ choice (English, isiZulu, isiXhosa, SeSotho, and Afrikaans). Data was entered and stored on a secure online research electronic data capture repository (REDCap, version 10.6.14, Vanderbilt University, Nashville, TN, USA) hosted by the University of Oxford (Oxford, UK) on behalf of ISARIC.

### Statistical analysis

As we did not base the study upon underlying data, the sample size was based purely on enrolment capability and resources available at the time.

We report here the prevalence of symptoms at 1 month and 3 months follow-up and present a detailed analysis of PCC at 3 months from hospital discharge. We used frequencies and percentages to summarise categorical data, and continuous data were expressed as median (IQR). We used frequency distribution tables and graphs to describe demographics, the prevalence of symptoms, comorbidities, and changes in health and occupational status.

We implemented five negative binomial regression models to estimate factors associated with the following outcomes at 3 months from hospital discharge: new or persistent symptoms, self-reported non-recovery, new or worsening breathlessness, new or worsening disability, and depression or anxiety (a description of how outcome variables were defined is presented in the appendix, p 1). Because of the very scarce knowledge of the pathophysiology of PCC at the time of writing, the model was not intended to assess cause–effect relationships, but rather to explore factors associated with PCC. As more evidence becomes available, the use of direct acyclic graphs to assess potential causality of the measured variables will be considered in future analyses.

Variables measured for association with the different outcomes in the multivariable models were age, sex, ethnicity, presence of individual comorbid conditions (asthma, diabetes, hypertension, chronic cardiac disease, chronic kidney disease, malignancy, tuberculosis, HIV, and obesity), number of symptoms during acute infection, treatment in an intensive care unit (ICU), and treatment with oxygen or ventilation. Variables such as age, sex, and ethnicity were included in the model a priori. This was done in an effort to reduce possible confounding factors on the basis of known clinical plausibility with health outcomes. For each multivariable model, variables with p<0·2 in the univariable analysis were included for multivariable analysis. Manual backward elimination was implemented, and the final model selection was guided by minimisation of the Akaike information criterion or Bayesian information criterion. Statistical significance for the multivariable analysis was assessed at p<0·05. Statistical analyses were done with Stata, version 16.

### Role of the funding source

The funders of the study had no role in study design, data collection, data analysis, data interpretation, or writing of the report.

## Results

Of the 241 159 COVID-19 admissions reported to DATCOV between Dec 1, 2020, and Aug 23, 2021, 124 174 were eligible for inclusion, and 8309 were randomly selected for enrolment. Of the 3094 patients that we were able to contact, 2410 (77·9%) consented to participate in the study (figure). The enrolled participants and those who were not enrolled in the study had similar demographic characteristics and distribution of comorbidities, except that they differed by the health sector they were hospitalised in (more patients admitted in the private sector were included in the study than those admitted to public hospitals; appendix p 2).

**Figure:**
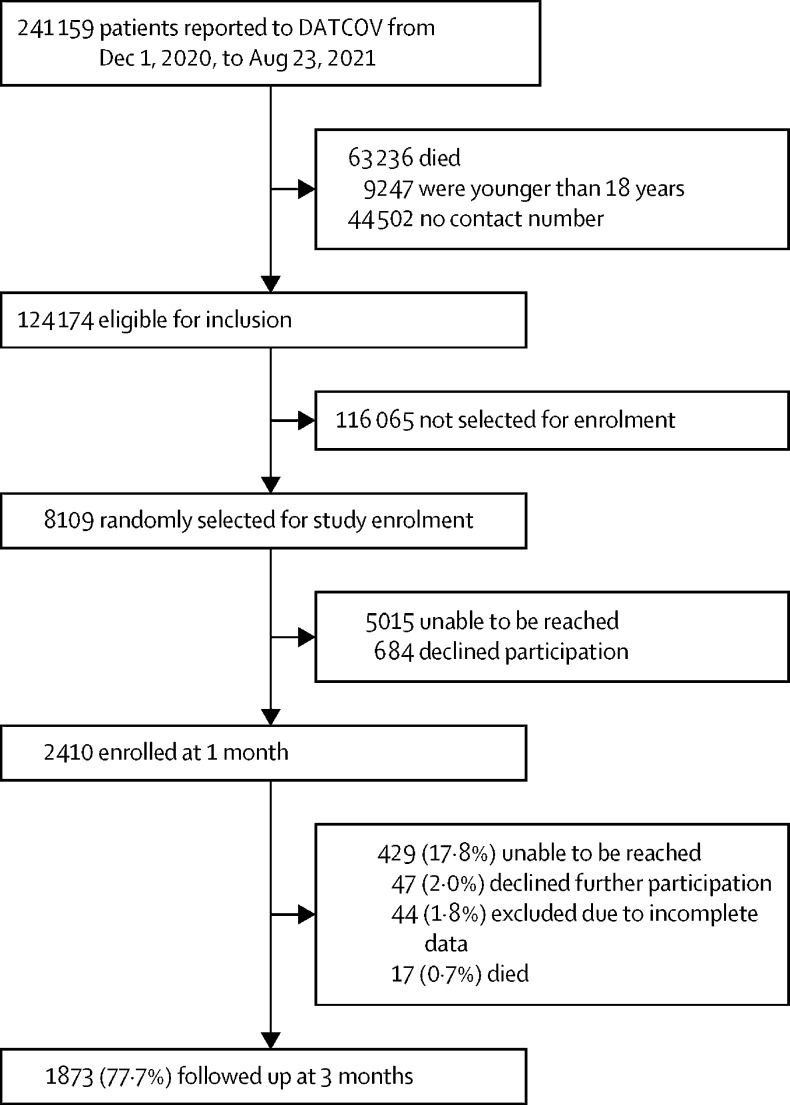
Study population based on inclusion criteria

Among 1873 participants followed up at approximately 3 months, the median time between hospital discharge and telephone follow-up was 95 days (IQR 84–126). Of the 1873 participants, 960 (53·1%) were women and 913 (48·8%) were men, the median age was 52 years (41–62), and 1102 (58·8%) were aged 40–64 years. 896 (47·8%) participants were Black, 677 (36·2%) were White, 154 (8·2%) were Mixed race and 132 (7·1%) were Indian (table 1). More than two thirds of participants had at least one comorbid condition (1300 [69·4%]). The most common self-reported comorbidities were hypertension, obesity, and diabetes; HIV was reported by 95 (5·1%) participants. 462 (24·7%) participants were hospitalised in the public sector. Most participants (1324 [70·7%]) received supplemental oxygen therapy during hospitalisation, whereas 612 (32·7%) required admission to ICU and 179 (9·6%) received invasive mechanical ventilation (table 1).

**Table 1:** Characteristics of participants at 3 months follow-up, South Africa

		**Female**	**Male**	**Total**
Participants		960 (51·3%)	913 (48·8%)	1873 (100%)
Age, years		50 (38–60)	53 (44–63)	52 (41–62)
Age group, years				
	<40	257 (26·8%)	150 (16·4%)	407 (21·7%)
	40–64	539 (56·2%)	563 (61·7%)	1102 (58·8%)
	≥65	164 (17·1%)	200 (21·9%)	364 (19·4%)
Ethnicity				
	Black	515 (53·6%)	381 (41·7%)	896 (47·8%)
	White	306 (31·9%)	371 (40·6%)	677 (36·1%)
	Mixed	73 (7·6%)	82 (9·0%)	154 (8·2%)
	Indian	61 (6·4%)	71 (7·8%)	132 (7·0%)
	Asian or other	0	1 (0·1%)	1 (0·1%)
	Unknown	5 (0·5%)	8 (0·9%)	13 (0·7%)
Pregnant		31 (3·2%)	NA	31 (1·7%)
Number of comorbidities				
	None	295 (30·7%)	278 (30·4%)	573 (30·6%)
	1	303 (31·6%)	310 (34·0%)	613 (32·7%)
	2	234 (24·4%)	196 (21·5%)	430 (23·0%)
	≥3	128 (13·3%)	129 (14·1%)	257 (13·7%)
Comorbidity and risk factors				
	Hypertension	330 (34·4%)	339 (37·1%)	669 (35·7%)
	Obesity	244 (25·4%)	230 (25·2%)	474 (25·3%)
	Diabetes	204 (21·3%)	214 (23·4%)	418 (22·3%)
	Heart disease	47 (4·9%)	64 (7·0%)	111 (5·9%)
	High cholesterol	43 (4·5%)	61 (6·7%)	104 (5·6%)
	HIV	67 (7·0%)	28 (3·1%)	95 (5·1%)
	Asthma	58 (6·0%)	34 (3·7%)	92 (4·9%)
	Kidney disease	11 (1·1%)	19 (2·1%)	30 (1·6%)
	Rheumatological disorder	18 (1·9%)	9 (1·0%)	27 (1·4%)
	Cancer	14 (1·5%)	12 (1·3%)	26 (1·4%)
	Chronic lung disease	11 (1·1%)	14 (1·5%)	25 (1·3%)
	Thyroid disease	20 (2·1%)	4 (0·4%)	24 (1·3%)
	Depression	11 (1·1%)	10 (1·1%)	21 (1·1%)
	Other	126 (13·1%)	97 (10·6%)	223 (11·9%)
Health sector				
	Private	687 (71·6%)	724 (79·3%)	1411 (75·3%)
	Public	273 (28·4%)	189 (20·7%)	462 (24·7%)
Received supplemental oxygen in hospital		650 (67·7%)	674 (73·8%)	1324 (70·7%)
Admitted to ICU		265 (27·6%)	347 (38·0%)	612 (32·7%)
Received invasive mechanical ventilation		75 (7·8%)	104 (11·4%)	179 (9·6%)
Received at least one dose of a COVID-19 vaccine		478 (49·8%)	464 (50·8%)	942 (50·3%)

Data are n (%) or median (IQR). ICU=intensive care unit. NA=not applicable.

Of the 1873 participants followed up at 3 months after hospital discharge, 1672 (89·3%) reported symptoms during the acute phase of their COVID-19 illness (table 2). The median number of acute symptoms reported was four (2–7). Among participants reporting acute symptoms, 142 (7·6%) reported one, 211 (11·3%) reported two, 247 (13·2%) reported three, and 1072 (57·2%) reported four or more symptoms. The most commonly reported symptoms on hospital admission during the acute COVID-19 illness were fatigue or malaise, shortness of breath, fever, cough, and headache (table 2). The most commonly reported symptoms at 1 month were fatigue or malaise, shortness of breath, headache, weakness of the arms or legs, and confusion or lack of concentration.

**Table 2:** Prevalence of acute and post-COVID-19 symptoms reported by 1873 participants at 1 month and 3 months after discharge from hospital, South Africa

	Acute*COVID-19	**1 month follow-up**	**3 months follow-up**
No symptoms	201 (10·7%)	370 (19·8%)	624 (33·3%)
Fatigue or malaise	1063 (56·8%)	1226 (65·5%)	942 (50·3%)
Shortness of breath	942 (50·3%)	864 (46·1%)	439 (23·4%)
Confusion or lack of concentration	NR	318 (17·0%)	327 (17·5%)
Headache	696 (37·2%)	406 (21·7%)	258 (13·8%)
Problems seeing or blurred vision	NR	224 (12·0%)	190 (10·1%)
Joint pain (arthralgia)	656 (35·0%)	145 (7·7%)	175 (9·3%)
Muscle aches (myalgia)	40 (2·1%)	198 (10·6%)	156 (8·3%)
Any other symptoms	170 (9·1%)	148 (7·9%)	149 (8·0%)
Chest pain	475 (25·4%)	280 (14·9%)	132 (7·0%)
Dizziness or light-headedness	45 (2·4%)	222 (11·9%)	116 (6·2%)
Dry cough	849 (45·3%)	276 (14·7%)	78 (4·2%)
Loss of taste	111 (5·9%)	162 (8·6%)	51 (2·7%)
Abdominal pain	154 (8·2%)	97 (5·2%)	44 (2·3%)
Loss of smell	153 (8·2%)	123 (6·6%)	44 (2·3%)
Back pain or backache	33 (1·8%)	34 (1·8%)	43 (2·3%)
Cough with sputum	161 (8·6%)	94 (5·0%)	40 (2·1%)
Diarrhoea	293 (15·6%)	73 (3·9%)	38 (2·0%)
Skin rash	54 (2·9%)	73 (3·9%)	35 (1·9%)
Fever	866 (46·2%)	43 (2·3%)	29 (1·5%)
Nausea or vomiting	240 (12·8%)	43 (2·3%)	25 (1·3%)
Nasal congestion or sinusitis	21 (1·1%)	9 (0·5%)	17 (0·9%)
Bleeding	29 (1·5%)	24 (1·3%)	11 (0·6%)
Loss of appetite or anorexia	429 (22·9%)	12 (0·6%)	10 (0·5%)
Body pain or body ache	15 (0·8%)	0	2 (0·1%)
Seizures	16 (0·9%)	4 (0·2%)	2 (0·1%)

Data are n (%). NR=not reported.

* Symptoms reported before hospital admission, upon admission, or both, irrespective of the reason for admission.

3 months after hospital discharge, 1249 (66·7%) participants reported new or persistent symptoms. Among these, 371 (19·8%) reported one, 277 (14·8%) two, 180 (9·6%) three, and 421 (22·5%) four or more persistent symptoms. The most commonly reported symptoms 3 months post-hospital discharge were fatigue, shortness of breath, confusion or lack of concentration, headache, and problems seeing or blurred vision.

Comparing symptoms at 1 month with those at 3 months after hospital discharge, we observed a decline in the prevalence of persistent symptoms, from 1978 (82·1%) of 2410 participants reporting at least one symptom at 1 month to 1249 (66·7%) of 1873 at 3 months. Fatigue, malaise, myalgia, light-headedness, and loss of taste were more commonly reported at 1 month than during the acute COVID-19 phase; arthralgia and back pain were more commonly reported at 3 months than at 1 month. Acute infection symptoms such as fever, cough, and shortness of breath were less common at 1 month and 3 months than during the acute COVID-19 phase.

Most participants reported no problems with mobility, self-care, doing usual activities, pain or discomfort, or anxiety or depression at the 3-month follow-up assessment (table 3). However, 219 (11·7%) reported problems with mobility, 255 (13·6%) with doing their usual activities including work, study, housework, and family or leisure activities, and 87 (4·6%) with self-care, which were all similar for men and women (table 3).

**Table 3:** Effect of persistent symptoms after COVID-19 on activities of daily living, South Africa

		**Female (n=960 [51·3%])**	**Male (n=913 [48·8%])**	**Total (n=1873)**	**p value**
Mobility		..	..	..	0·16
	No problems	827 (86·1%)	823 (90·1%)	1650 (88·1%)	..
	Slight problems	89 (9·3%)	59 (6·5%)	148 (7·9%)	..
	Moderate problems	22 (2·3%)	18 (2·0%)	40 (2·1%)	..
	Severe problems	10 (1·0%)	5 (0·5%)	15 (0·8%)	..
	Unable	10 (1·0%)	6 (0·7%)	16 (0·9%)	..
	Missing	2 (0·2%)	2 (0·2%)	4 (0·2%)	..
Self-care		..	..	..	0·62
	No problems	906 (94·4%)	876 (95·9%)	1782 (95·1%)	..
	Slight problems	34 (3·5%)	25 (2·7%)	59 (3·2%)	..
	Moderate problems	6 (0·6%)	4 (0·4%)	10 (0·5%)	..
	Severe problems	6 (0·6%)	2 (0·2%)	8 (0·4%)	..
	Unable	6 (0·6%)	4 (0·4%)	10 (0·5%)	..
	Missing	2 (0·2%)	2 (0·2%)	4 (0·2%)	..
Usual activities		..	..	..	0·16
	No problems	812 (84·6%)	803 (88·0%)	1615 (86·2%)	..
	Slight problems	96 (10·0%)	76 (8·3%)	172 (9·2%)	..
	Moderate problems	33 (3·4%)	21 (2·3%)	54 (2·9%)	..
	Severe problems	8 (0·8%)	8 (0·9%)	16 (0·9%)	..
	Unable	10 (1·0%)	3 (0·3%)	13 (0·7%)	..
	Missing	1 (0·1%)	2 (0·2%)	3 (0·2%)	..
Pain or discomfort		..	..	..	0·0006
	No pain	756 (78·8%)	784 (85·9%)	1540 (82·2%)	..
	Slight pain	137 (14·3%)	87 (9·5%)	224 (12·0%)	..
	Moderate pain	43 (4·5%)	33 (3·6%)	76 (4·1%)	..
	Severe pain	21 (2·2%)	5 (0·5%)	26 (1·4%)	..
	Extreme pain	1 (0·1%)	1 (0·1%)	2 (0·1%)	..
	Missing	2 (0·2%)	3 (0·3%)	5 (0·3%)	..
Anxiety or depression		..	..	..	<0·0001
	No anxiety or depression	720 (75·0%)	766 (83·9%)	1486 (79·3%)	..
	Slight anxiety or depression	122 (12·7%)	100 (11·0%)	222 (11·9%)	..
	Moderate anxiety or depression	81 (8·4%)	31 (3·4%)	112 (6·0%)	..
	Severe anxiety or depression	25 (2·6%)	12 (1·3%)	37 (2·0%)	..
	Extreme anxiety or depression	8 (0·8%)	3 (0·3%)	11 (0·6%)	..
	Missing	4 (0·4%)	1 (0·1%)	5 (0·3%)	..

Activities of daily living measured with the EuroQol Research Foundation EQ-5D tool, version 2.1.

At 3 months, 328 (17·5%) participants reported having slight, moderate, severe, or extreme pain. More women (202 [21·0%]) than men (126 [13·8%]) reported having pain (p=0·0006). 222 (11·9%) participants reported mild anxiety or depression, 112 (6·0%) reported moderate anxiety or depression, and 48 (2·6%) reported having severe to extreme anxiety or depression. More women (236 [24·6%]) than men (146 [16·0%]) reported having anxiety or depression (p<0·0001; table 3).

Most participants reported no change in their ability to see, hear, walk, remember, and self-care before having COVID-19 using the UN–Washington score. However, 409 (21·8%) reported worsening ability to remember, 268 (14·3%) reported worsening ability to walk, and 179 (9·6%) reported worsening ability to see (appendix p 2).

Among 1837 participants followed up at 3 months, 837 (44·7%) consulted with a general practitioner or primary health-care clinic, and 82 (4·4%) were readmitted to hospital after hospital discharge. 36 (1·9%) participants reported to still be on domiciliary supplemental oxygen 3 months after hospital discharge, and the median time on home oxygen was 21 days (7–30).

Among all participants, 1099 (58·7%) were working full time before contracting COVID-19, whereas 377 (20·1%) were retired, 227 (12·1%) were unemployed, and 69 (3·7%) were working part-time. 47 (2·5%) participants reported a change in occupation at the 3 month assessment, including being retrenched, medically boarded, or required to reduce working hours. Of these, 15 (31·9%) attributed the changes in occupation to the effects of PCC. Participants were surveyed about their social habits before and after the COVID-19 diagnosis: 81 (24·4%) of 332 self-reported smokers were smoking less, and 468 (65·7%) of 712 self-reported alcohol consumers were consuming less alcohol. Additionally, 1065 (56·9%) were eating healthier food and 582 (31·1%) were exercising more, whereas 453 (24·2%) were exercising less.

On multivariate analysis, the factors associated with new or persistent symptoms were being female (adjusted incident rate ratio 1·20, 95% CI 1·04–1·38) and ICU admission (1·17, 1·01–1·37; table 4). Factors associated with persistent breathlessness after acute COVID-19 illness were being female, White or Mixed ethnicity compared with Black ethnicity, receiving supplemental oxygen during admission, and reporting four or more acute symptoms compared with no symptoms during the acute COVID-19 phase (table 4). Factors associated with self-reported non-recovery were being female; White, Mixed, and Indian compared with Black ethnicity; receiving supplemental oxygen during admission; receiving mechanical ventilation during admission; and pre-existing asthma, type 1 diabetes, hypertension, and depression. The underlying comorbidity of type 1 diabetes was protective against self-reported non-recovery (table 4). Factors associated with new or worse disability were age 40–64 years and age 65 years or older compared with age younger than 40 years, being female, receiving supplemental oxygen during admission, ICU admission, and presence of one to three acute symptoms and four or more acute symptoms compared with no symptoms during acute COVID-19 illness (table 4). Factors associated with anxiety or depression 3 months after hospital discharge included being female, age 65 years or older, and four or more acute symptoms compared with no symptoms during acute COVID-19 illness. Being 65 years or older compared with younger than 40 years was protective against anxiety or depression (table 4).

**Table 4:** Multivariate analyses of factors associated with five different post-COVID-19 condition outcomes, South Africa, Dec 1, 2020, to Aug 23, 2021 (n=1873)

	**New or persistent symptoms**	**Breathlessness**	**Self-reported non-recovery**	**New or worsening disability**	**Anxiety or depression**
**Age group, years**					
<40	1 (ref)	1 (ref)	1 (ref)	1 (ref)	1 (ref)
40–64	1·04 (0·86–1·27)	1·04 (0·71–1·54)	0·82 (0·51–1·31)	1·30 (1·03–1·65)*	0·98 (0·74–1·29)
≥65	1·14 (0·89–1·46)	0·91 (0·56–1·47)	0·58 (0·32–1·07)	1·37 (1·03–1·80)*	0·67 (0·45–0·99)*
**Sex**					
Male	1 (ref)	1 (ref)	1 (ref)	1 (ref)	1 (ref)
Female	1·20 (1·04–1·38)*	1·37 (1·05–1·77)*	1·92 (1·37–2·70)*	1·43 (1·21–1·67)*	1·55 (1·25–1·92)*
**Ethnicity**					
Black	1 (ref)	1 (ref)	1 (ref)	1 (ref)	1 (ref)
White	1·08 (0·92–1·28)	1·86 (1·32–2·63)*	1·84 (1·24–2·70)*	1·18 (0·99–1·41)	1·08 (0·85–1·39)
Mixed	1·22 (0·95–1·56)	1·69 (1·04–2·76)*	1·95 (1·10–3·45)*	1·15 (0·78–1·69)	1·03 (0·70–1·50)
Indian	1·14 (0·88–1·48)	1·29 (0·73–2·26)	1·89 (1·02–3·52)*	0·92 (0·60–1·39)	0·78 (0·50–1·23)
Unknown	1·15 (0·59–2·25)	1·21 (0·29–5·00)	NA	2·04 (1·01–4·13)*	1·32 (0·49–3·60)
**Admitted to ICU**					
No	1 (ref)	1 (ref)	1 (ref)	1 (ref)	1 (ref)
Yes	1·17 (1·01–1·37)*	1·31 (0·99–1·73)	1·09 (0·74–1·59)	1·29 (1·08–1·53)*	1·01 (0·74–1·39)
**Received supplemental oxygen**					
No	1 (ref)	1 (ref)	1 (ref)	1 (ref)	1 (ref)
Yes	1·12 (0·94–1·34)	2·19 (1·41–3·39)*	1·52 (0·97–2·38)	1·34 (1·09–1·65)*	1·08 (0·86–1·36)
**Received invasive mechanical ventilation**					
No	1 (ref)	1 (ref)	1 (ref)	1 (ref)	1 (ref)
Yes	1·02 (0·80–1·32)	1·21 (0·80–1·85)	2·07 (1·28–3·32)*	1·09 (0·84–1·41)	1·08 (0·70–1·65)
**Comorbidities**					
Asthma	NA	1·42 (0·91–2·25)	2·19 (1·35–3·57)*	1·32 (0·98–1·79)	NA
Obesity	1·10 (0·96–1·27)	1·26 (0·97–1·64)	NA	NA	1·20 (0·97–1·49)
Diabetes type 1	NA	NA	0·64 (0·41–0·99)*	NA	NA
Diabetes type 2	NA	NA	NA	NA	NA
Hypertension	NA	NA	1·81 (1·28–2·56)*	NA	NA
Rheumatological disorder	NA	NA	NA	1·60 (0·98–2·59)	NA
Depression	NA	NA	2·37 (1·02–5·48)*	NA	NA
**Acute COVID-19 symptoms**					
None	1 (ref)	1 (ref)	1 (ref)	1 (ref)	1 (ref)
1–3	1·12 (0·81–1·53)	2·44 (0·98–6·09)	1·22 (0·52–2·92)	1·47 (1·01–2·13)*	1·07 (0·63–1·80)
≥4	1·25 (0·92–1·69)	2·73 (1·11–6·71)*	1·81 (0·82–3·99)	1·64 (1·15–2·35)*	1·76 (1·08–2·85)*

Data are adjusted incidence rate ratio (95% CI). ICU=intensive care unit. NA=not applicable.

* Significant association.

## Discussion

We report that new or persistent symptoms were present in two-thirds of participants with confirmed SARS-CoV-2 infection 3 months post-hospital discharge in this large cohort in South Africa. To our knowledge, this is the first study of this size that describes the prevalence and risk factors for PCC in South Africa.

Our estimates are similar to those of other studies, which reported prevalence ranging from 50% to 78%.^13^, ^14^, ^15^, ^16^ Similar to other published studies, the most common PCC symptoms reported by individuals who had COVID-19 in our cohort were fatigue, shortness of breath, confusion or lack of concentration, headache, and problems seeing or blurred vision. Studies with shorter follow-up (≤2 months) reported higher frequency of acute symptoms such as cough, fever, and acute gastrointestinal symptoms, which might indicate persistent infection,^17^, ^18^, ^19^, ^20^ whereas studies with follow-up beyond 3 months reported fatigue, shortness of breath, and musculoskeletal symptoms more frequently.^13^, ^14^, ^15^, ^16^, ^21^, ^22^, ^23^, ^24^

Few studies have reported risk factors for PCC. In this study, we identified an association of PCC with age, sex, ethnicity, number of acute COVID-19 symptoms, comorbidities, and acute COVID-19 severity (ICU stay, oxygen use, and invasive mechanical ventilation). Older adults (aged 65 years or older) had an increased risk of new or worsening disability and new or persistent symptoms. This association might result from increased comorbid disease, poorer overall health status, and relative exertion intolerance.^25^ There is conflicting evidence in the literature on age as a risk factor for PCC. Some studies found an association between age younger than 65 years and persistence of symptoms,^20^, ^26^, ^27^ some reported that increasing age was a risk factor,^24^, ^28^ and others did not find an association between age and PCC.^29^

Being female was identified as a risk factor for all outcomes when compared with being male. Being female has been found to have a significant association with PCC in other studies,^26^, ^29^, ^30^, ^31^, ^32^, ^33^ including associations with fatigue and breathlessness,^32^ incomplete recovery, and greater disability.^26^, ^34^ It is important to consider the effect of survival bias in the case of PCC. Men have a higher risk of mortality during the acute COVID-19 phase than women,^35^ but being male has not been found to be a significant factor associated with PCC,^29^ suggesting that women, typically of younger age, are more likely to survive acute disease, but subsequently have worse long-term outcomes.^34^ Additionally, health-seeking behaviour in men is lower than in women, and men might be less inclined to report persistence of poor health.^34^ However, other factors such as the effect of sex hormones on the risk of developing PCC, socioeconomic effects of COVID-19, and health access disparities need further exploration.

Our study showed a significant association between non-Black ethnicity and PCC outcomes of breathlessness and self-reported non-recovery in South Africa. A meta-analysis^33^ including ten studies based predominantly in Europe also found that Black and South Asian ethnicity were protective against PCC. There is scarce data available on associations of ethnicity with PCC, and the reasons for differences in persistent symptoms by ethnicity is unclear and will require further investigation.

Admission to ICU was significantly associated with persistent symptoms and new or worsening disability. Post-intensive care syndrome (PICS) has been previously described as a collection of physical, mental, and emotional post-ICU persistent sequelae.^36^, ^37^, ^38^, ^39^, ^40^ The PCC persistent symptoms identified among individuals who were admitted to ICU might overlap with or be further compounded by PICS. Participants who received supplemental oxygen during hospital admission also showed significant associations with breathlessness and new or worsening disability, whereas mechanical ventilation during admission only showed significant association with self-reported non-recovery. This is in keeping with the outcomes in a study^26^ that found that participants who had received invasive ventilation were nearly four times more likely to report incomplete recovery than those who had not required any supplemental ventilation or oxygen.

Pre-existing comorbidities such as asthma, type 1 diabetes, hypertension, and depression were associated with PCC. Previous studies have also found an increased risk of PCC in people with some pre-existing comorbidities^28^, ^33^, ^34^ such as asthma,^29^, ^33^ hypertension,^28^ obesity,^28^, ^33^ neuro-psychological conditions,^28^, ^33^ and immunosuppressive conditions.^28^

The pathophysiology of PCC is not well understood, with multiple hypotheses attempting to describe the potential mechanisms on the basis of previous coronavirus outbreaks and other RNA viruses. Some have suggested that PCC might be the result of tissue or organ damage^41^, ^42^ or inflammatory and immune pathway dysfunction (including chronic inflammation, hyperactive immune cells, and autoimmunity as a result of molecular mimicry).^41^, ^42^ Additionally, it is theorised that PCC could be due to viral persistence,^41^ reactivation of latent pathogens (ie, herpesviruses or Epstein-Barr virus),^41^ or disruption of commensal microbiome or virome communities.^42^ Other plausible mechanisms include clotting or coagulation issues and dysfunctional brainstem or vagus nerve signalling,^42^ as well as vasculitis in major arterial vessels.^43^ It is plausible that patients with underlying comorbidities admitted with COVID-19 would be at greater risk of PCC because of pre-existent tissue damage and more severe COVID-19 illness associated with longer hospital stays, more complications, and the likelihood of receiving oxygen, invasive ventilation, steroids, and other treatments. Unfortunately, we did not collect data on chronic medications and were unable to draw any conclusions that would suggest that chronic medications and their related side-effects played a role in the persistence of symptoms, although we acknowledge that this might be a confounding factor.

Participants with more symptoms during the acute COVID-19 phase were more likely to report breathlessness, new or worsening disability, and anxiety or depression. This is in line with a study^29^ that found that participants who presented with more than five symptoms in the first week of acute illness had a significantly increased risk of PCC.

We report a decline in the prevalence of PCC between the 1-month and 3-month assessment (82% to 67%), consistent with other studies.^4^ A longitudinal study^44^ of patients who were previously hospitalised with COVID-19 found that the prevalence of new or persistent symptoms declined from 68% at 6 months to 49% at 12 months, suggesting a steady resolution of symptoms with time. However, a long recovery time might still have a substantial effect on the individual and families, as well as wider socioeconomic and public health implications.

Our study was a large, nationally representative longitudinal cohort study and, to our knowledge, the first of its kind in South Africa and Africa. As part of the global ISARIC collaboration, we used standardised and validated tools, which allow comparison across participating countries. The alignment to the DATCOV hospital surveillance system and national SARS-CoV-2 case list allowed us to identify potential participants and to link key demographic and clinical data related to their hospital admission. These are preliminary results of an ongoing study. The study will continue to follow up participants until 12 months after hospital discharge.

The study had several limitations. First, the study was limited to participants who were hospitalised with SARS-CoV-2. It would be of interest to include controls with other respiratory infections who tested negative for SARS-CoV-2 to understand the effect of COVID-19 on continuing morbidity, but these data are not available in this study and a case-control analysis would be ideal. However, our study did recruit a cohort of patients with SARS-CoV-2 infection who were not hospitalised, and future analysis will compare hospitalised and non-hospitalised cohorts. This will help to distinguish between symptoms related to PICS and complications arising directly from hospitalisation. Second, participants who had symptoms might have been more likely to participate than those who did not. The possibility of recall and response bias and the subjective rating of symptoms might affect the reporting. At the time the follow-up survey was done, patients were not actively tested for SARS-CoV-2, and we did not exclude the possibility of acute COVID-19 re-infection; however, participants were screened by use of the questionnaire as to whether they had recently tested for SARS-CoV-2. Additionally, the study did not attempt to investigate the cause of the persistent symptoms or to rule out other causes for persistent symptoms because we sought only to report the presence of persistent symptoms in patients previously hospitalised with COVID-19. Third, and last, all participants were enrolled through a telephone assessment, limiting the enrolment to those who had a telephone number recorded. This might explain the greater proportion of individuals from higher socioeconomic strata that were enrolled and the resultant sampling bias. We had a lower response rate than anticipated at the 1 month enrolment (2413 [29·7%] of 8112) because of distrust of the study's legitimacy, privacy concerns, time constraints, and incorrect contact numbers available. Of the 2413 participants recruited at 1 month, the retention rate at 3 months was 1873 (77·6%) because of efforts to explain the importance of the study to enrolled participants. However, our comparison of demographic characteristics of participants and of those admitted to hospital from which the sample was drawn found similar distribution by sex, ethnicity, and province, except for a higher proportion of study participants from the private sector, aged 40–64 years (related to availability of contact details and willingness to participate), and with more severe acute illness (related to under-reporting in the DATCOV system; appendix p 2).

The findings of this national study show a high prevalence of persistent symptoms at 3 months after hospital discharge following SARS-CoV-2 infection, with effects on daily living, functioning, and occupation. The high burden of PCC is concerning for South Africa, from an individual and public health perspective, because of the potential additional burden on an already overwhelmed health-care system, reduced work productivity, and increased need for economic support. These findings can inform public health measures, including identifying individuals at increased risk of developing PCC, and providing patient support and information. Moreover, the findings can inform development of clinical pathways and guidelines for the care of these patients and health service planning. Long-term follow-up of this cohort will provide further insights into the evolution of PCC in South Africa. Future analyses will also allow us to assess the effect of COVID-19 vaccination on persistent symptoms and to compare PCC between South Africa's first, second, third, and fourth pandemic waves, when different virus variants predominated.

## Data sharing

Restrictions apply to the availability of these data and so they are not publicly available. However, data can be made available from the corresponding author upon reasonable request and with the permission of the South African Department of Health, the Bill & Melinda Gates Foundation, and NICD.

## Declaration of interests

We declare no competing interests.
