# Supplementary material for: Post-COVID-19 condition 3 months after hospitalisation with SARS-CoV-2 in South Africa: a prospective cohort study
Source: Lancet Glob Health. 2022 Aug 9;10(9):e1247–56. doi: 10.1016/S2214-109X(22)00286-8 (PMC9363040; doi:10.1016/S2214-109X(22)00286-8)
Supplement: Supplementary appendix [file mmc1.pdf]

THE LANCET
Global Health

Supplementary appendix

This appendix formed part of the original submission and has been peer reviewed. We post it as supplied by the authors.

Supplement to: Dryden M, Mudara C, Vika C, et al. Post-COVID-19 condition 3 months after hospitalisation with SARS-CoV-2 in South Africa: a prospective cohort study. *Lancet Glob Health* 2022; **10:** e1247–56.

Supplemental Material

Supplementary Methods: Statistical Analysis

The outcome variables were obtained as follows:

- New or persistent symptoms: obtained from the number of persistent symptoms the participants have. Those who did not have any symptoms were classified as “No persistent symptoms” and those with 1 or more symptoms were classified as “New or persistent symptoms”.
- Self-reported non-recovery: obtained from the Likert scale question; “Do you feel fully recovered from your COVID-19?”. The five possible responses to this question were collapsed into two categories “Recovered” and “Not recovered”. The response “Neither agree or disagree” was treated as neutral and excluded from the analysis.
- New or worsening breathlessness: obtained from the question “How breathless you feel TODAY and how breathless you felt BEFORE your COVID-19 illness”. Responses were coded from a value of 1 to 5 representing less severe to very severe breathlessness. The output obtained from difference between breathlessness before and breathlessness after (breathless before – breathless after) was classified into two categories. A difference greater or equal to zero was classified as “No new or worsening breathlessness” = 0 (breathing better or no change in breathlessness) and a difference of less than zero was classified as “New or worsening breathlessness” = 1.
- New/worse disability: constructed from the Washington short set tool (13) which includes questions on changes in vision, hearing, mobility, remembering, self-care and communication (comparing before and after contracting COVID-19). Responses to these questions were coded from 1 to 4 representing no difficulty to inability to function. Change in any disability was classified as “Better”, “No Change” and “Worse/New disability”. The outcome variable “New/worse disability” was constructed if worse change or a new disability was noted in any one of the stated Washington disability categories (“No disability” = 0 and “At least one disability” = 1).
- Anxiety/Depression: constructed from the EuroQol Research Foundation EQ-5D™ tool v2.1 (11). The responses to the Likert scale Anxiety/Depression question were collapsed into two categories, “anxiety/ depression” if there was any level of anxiety or depression reported and “no anxiety/ depression”.

Supplementary Table 1. Prevalence of acute COVID-19 symptoms reported by 1873 participants before hospital admission, upon admission, or both, irrespective of the reason for admission.

**Characteristic**	**Acute COVID-19**						
	**Female**		**Male**		**Total**		**P Value**
	**Number**	**Percentage**	**Number**	**Percentage**	**Number**	**Percentage**	
	**960**	**51.3%**	**913**	**48.7%**	**1873**		
**Symptoms**							
Abdominal Pain	82	8.5	72	7.9	154	8.2	0.90
Altered Consciousness	127	13.2	110	12.1	237	12.7	0.62
Back Pain/Back Ache	17	1.8	16	1.8	33	1.8	0.98
Bleeding (Haemorrhage)	18	1.9	11	1.2	29	1.6	0.15
Body Pain/Body Ache	6	0.6	9	1.0	15	0.8	0.38
Chest Pain	256	26.7	219	24.0	475	25.4	0.25
Chills	9	0.9	8	0.9	17	0.9	0.89
Conjunctivitis	43	4.5	35	3.8	78	4.2	0.91
Cough	431	44.9	418	45.8	849	45.3	0.23
Cough With Haemopytsis	21	2.2	22	2.4	43	2.3	0.89
Cough With Sputum Production	79	8.2	82	9.0	161	8.6	0.83
Diarrhoea	154	16.0	139	15.2	293	15.6	0.06
Dizziness	29	3.0	16	1.8	45	2.4	0.07
Fatigue / Malaise	551	57.4	512	56.1	1063	56.8	0.45
Fever	416	43.3	450	49.3	866	46.2	0.01
Headache	371	38.7	325	35.6	696	37.2	0.39
Inability To Walk	124	12.9	99	10.8	223	11.9	0.42
Joint Pain (Arthralgia)	227	23.7	202	22.1	429	22.9	0.40
Loss of Appetite/Anorexia	76	7.9	77	8.4	153	8.2	0.68
Loss of Smell	60	6.3	51	5.6	111	5.9	0.54
Loss of Taste	74	7.7	75	8.2	149	8.0	0.69
Lower Chest Wall In Drawing	47	4.9	59	6.5	106	5.7	0.37
Lymphadenopathy	24	2.5	16	1.8	40	2.1	0.66
Muscle Aches (Myalgia)	327	34.1	329	36.0	656	35.0	0.56
Nasal Congestion/Sinusitis	12	1.3	9	1.0	21	1.1	0.59
Runny Nose (Rhinorrhoea)	105	10.9	151	16.5	256	13.7	0.00
Seizures	5	0.5	1	1.2	16	0.9	0.07
Shortness of Breath	474	49.4	468	51.3	942	50.3	0.20
Skin Rash	32	3.3	22	2.4	54	2.9	0.32
Skin Ulcers	10	1.0	8	0.9	18	1.0	0.39
Sore Throat	244	25.4	210	23.0	454	24.2	0.57
Sweating/ Sweating Profusely	15	1.6	9	1.0	24	1.3	0.27
Vomiting / Nausea	141	14.7	99	10.8	240	12.8	0.03
Wheezing	85	8.9	2	1.2	187	10.0	0.23
Any other new symptoms	96	10.00	74	8.11	170	9.08	0.280

Supplentary Table 2. Prevalence of acute COVID-19 symptoms reported by 1873 participants at 1 month follow up following discharge from hospital.

Characteristic	Post COVID-19 Symptoms at 1 month after hospital discharge						
	Female		Male		Total		P Value
	Number	Percentage	Number	Percentage	Number	Percentage	
	**960**	**51.3%**	**913**	**48.7%**	**1873**		
**Symptoms**							
Back Pain/Back Ache	23	2.4	11	1.2	34	1.8	0.05
Bleeding	11	1.2	13	1.4	24	1.3	0.55
Can't feel one side of the body or face	18	1.9	20	2.2	38	2.0	0.03
Can't fully move or control movement	45	4.7	34	3.7	79	4.2	0.02
Changes in menstruation*	35	3.7	0	0.0	35	1.9	0.00
Chest pains	162	16.9	118	12.9	280	15.0	0.01
Confusion/lack of concentration	187	19.5	131	14.4	318	17.0	0.00
Constipation	72	7.5	50	5.5	122	6.5	0.00
Diarrhoea	44	4.6	29	3.2	73	3.9	0.00
Dizziness/light headedness	125	13.0	97	10.6	222	11.9	0.03
Erectile dysfunction+	0	0.0	7	4.1	37	2.0	0.00
Fatigue	637	66.4	589	64.5	1226	65.5	0.28
Feeling sick/vomiting	28	2.9	15	1.6	43	2.3	0.04
Fever	24	2.5	19	2.1	43	2.3	0.16
Headache	241	25.1	165	18.1	406	21.7	0.00
Joint pain or swelling	90	9.4	55	6.0	145	7.7	0.00
Loss of Appetite/Anorexia	6	0.6	6	0.7	12	0.6	0.93
Loss of smell	69	7.2	54	5.9	123	6.6	0.04
Loss of taste	87	9.1	75	8.2	162	8.7	0.08
Lumps or rashes (purple/pink) on toes	8	0.8	6	0.7	14	0.8	0.05
Nasal Congestion/Sinusitis	6	0.6	3	0.3	9	0.5	0.35
Pain on breathing	67	7.0	53	5.8	120	6.4	0.03
Palpitations (heart racing)	114	11.9	67	7.3	181	9.7	0.00
Persistent cough - with phlegm	48	5.0	46	5.0	94	5.0	0.17
Persistent cough – dry	136	14.2	140	15.3	276	14.7	0.13
Persistent muscle pain	105	10.9	93	10.2	198	10.6	0.10
Problems passing urine	19	2.0	26	2.9	45	2.4	0.03
Problems seeing/blurred vision	125	13.0	99	10.8	224	12.0	0.02
Problems sleeping	163	17.0	114	12.5	277	14.8	0.02
Problems swallowing or chewing	20	2.1	16	1.8	36	1.9	0.08
Problems with Balance	102	10.6	76	8.3	178	9.5	0.01
Seizures/Fits	2	0.2	2	0.2	4	0.2	0.04
Shortness of breath/breathlessness	435	45.3	429	47.0	864	46.1	0.07
Skin Rash	34	3.5	39	4.3	73	3.9	0.26
Stomach/abdominal pain	62	6.5	35	3.8	97	5.2	0.01
Swollen ankles(s)	68	7.1	46	5.0	114	6.1	0.01
Tingling feeling/"pins and needles"	65	6.8	54	5.9	119	6.4	0.13
Weakness in arms or legs/muscle weakness	188	19.6	175	19.2	363	19.4	0.13
Any other new symptoms	76	7.92	72	7.89	148	7.90	0.717

Supplentary Table 3. Prevalence of acute COVID-19 symptoms reported by 1873 participants at 3 months follow up following discharge from hospital.

**Characteristic**	**Post COVID-19 Symptoms at 3 months after hospital discharge**						
	**Female**		**Male**		**Total**		**P Value**
	**Number**	**Percentage**	**Number**	**Percentage**	**Number**	**Percentage**	
	**960**	**51.3%**	**913**	**48.7%**	**1873**		
**Symptoms**							
Back Pain/Back Ache	31	3.2	12	1.3	43	2.3	0.01
Bleeding	5	0.5	6	0.7	11	0.6	0.52
Body Pain/Body Aches	2	0.2	0	0.0	2	0.1	0.17
Can't feel one side of the body or face	9	0.9	3	0.3	12	0.6	0.21
Can't fully move or control movement	20	2.1	10	1.1	30	1.6	0.35
Changes in menstruation*	23	2.4	0	0.0	23	1.2	0.00
Chest pains	80	8.3	52	5.7	132	7.1	0.05
Confusion/lack of concentration	209	21.8	118	12.9	327	17.5	0.00
Constipation	33	3.4	16	1.8	49	2.6	0.13
Diarrhoea	24	2.5	14	1.5	38	2.0	0.32
Dizziness/light headedness	78	8.1	38	4.2	116	6.2	0.00
Erectile dysfunction+	0	0.0	8	3.07	28	1.49	0.00
Fatigue	516	53.8	426	46.7	942	50.3	0.01
Feeling sick/vomiting	18	1.9	7	0.8	25	1.3	0.19
Fever	22	2.3	7	0.8	29	1.6	0.05
Hairloss	77	8.0	9	1.0	86	4.6	0.00
Headache	180	18.8	78	8.5	258	13.8	0.00
Joint pain or swelling	122	12.7	53	5.8	175	9.3	0.00
Loss of Appetite/Anorexia	6	0.6	4	0.4	10	0.5	0.58
Loss of smell	26	2.7	18	2.0	44	2.4	0.71
Loss of taste	26	2.7	25	2.7	51	2.7	0.59
Lumps or rashes (purple/pink) on toes	3	0.3	1	0.1	4	0.2	0.79
Nasal Congestion/Sinusitis	12	1.3	5	0.6	17	0.9	0.11
Pain on breathing	15	1.6	9	1.0	24	1.3	0.54
Palpitations (heart racing)	63	6.6	28	3.1	91	4.9	0.00
Persistent cough - with phlegm	23	2.4	17	1.9	40	2.1	0.80
Persistent cough – dry	49	5.1	29	3.2	78	4.2	0.17
Persistent muscle pain	105	10.9	51	5.6	156	8.3	0.00
Problems passing urine	6	0.6	8	0.9	14	0.8	0.52
Problems seeing/blurred vision	124	12.9	66	7.2	190	10.1	0.00
Problems sleeping	86	9.0	39	4.3	125	6.7	0.00
Problems swallowing or chewing	8	0.8	8	0.9	16	0.9	0.36
Problems with balance	42	4.4	24	2.6	66	3.5	0.26
Seizures/fits	1	0.1	1	0.1	2	0.1	0.36
Shortness of breath/breathlessness	232	24.2	207	22.7	439	23.4	0.45
Skin Rash	25	2.6	10	1.1	35	1.9	0.02
Stomach/abdominal pain	31	3.2	13	1.4	44	2.4	0.03
Swollen ankles(s)	48	5.0	16	1.8	64	3.4	0.00
Tingling feeling/"pins and needles"	46	4.8	26	2.9	72	3.8	0.16
Weakness in arms or legs/muscle weakness	85	8.9	60	6.6	145	7.7	0.11
Any other new symptoms	90	9.38	59	6.46	149	7.96	0.03

Supplementary Table 4. Characteristics of study patients and hospitalised patients in DATCOV, a hospital-based COVID-19 surveillance network coordinated by the National Institute for Communicable Diseases, South Africa.

	**Long COVID Study**			**DATCOV**		
	**Private**	**Public**	**Total**	**Private**	**Public**	**Total**
	1,411 (75.33%)	***462 (24.67%)***	***N=1873***	***91,231 (52.49%)***	***82,570 (47.51%)***	***N=173,801***
**Median age (IQR)**	53 [44 - 63]	44 [33 -58]	52 [41 - 62]	53 [42 - 63]	51 [36 - 63]	52 [39 - 63]
**Age group**						
<40 years	226 (16.0)	181 (39.2)	407 (21.7)	18,065 (19.8)	25,430 (30.8)	43,495 (25.0)
40-64 years	880 (62.4)	222 (48.1)	1,102 (58.8)	52,084 (57.1)	37,936 (45.9)	90,020 (51.8)
≥65 years	305 (21.6)	59 (12.8)	364 (19.4)	20,634 (22.6)	18,871 (22.9)	39,505 (22.7)
Unknown				448 (0.5)	333 (0.4)	781 (0.5)
**Sex**						
Female	687 (48.7)	273 (59.1)	960 (51.3)	47,537 (52.1)	50,186 (60.8)	97,723 (56.2)
Male	724 (51.3)	189 (40.9)	913 (48.8)	43,694 (47.9)	32,321 (39.1)	76,015 (43.7)
Unknown				-	63 (0.1)	63 (0.0)
**Ethnicity**						
Black	513 (36.4)	383 (82.9)	1,280 (53.1)	18,319 (20.1)	55,615 (67.4)	73,934 (42.5)
White	645 (45.7)	32 (6.9)	677 (36.2)	13,751 (15.8)	2,622 (3.2)	16,373 (9.4)
Mixed	120 (8.5)	34 (7.4)	154 (8.2)	4,212 (4.6)	3,842 (4.7)	8,054 (4.6)
Indian	119 (8.4)	13 (2.8)	132 (7.1)	4,934 (5.4)	1,306 (1.6)	6,240 (3.6)
Other/Asian	1 (0.1)	-	1 (0.1)	48 (0.1)	344 (0.4)	392 (0.2)
Unknown	13 (0.9)	-	13 (0.7)	49,967 (54.8)	18,841 (22.8)	68,808 (39.6)
**Province**						
Eastern Cape	62 (4.4)	36 (7.8)	98 (5.2)	5,069 (5.6)	8,752 (10.6)	13,821 (8.0)
Free State	110 (7.8)	44 (9.5)	154 (8.2)	4,847 (5.3)	4,659 (5.6)	9,506 (5.5)
Gauteng	588 (41.7)	144 (31.2)	732 (39.1)	35,272 (38.7)	21,736 (26.3)	57,008 (32.8)
KwaZulu-Natal	191 (13.5)	55 (11.9)	246 (13.1)	15,612 (17.1)	11,401 (13.8)	27,013 (15.5)
Limpopo	25 (1.8)	18 (3.9)	43 (2.3)	3,768 (4.1)	4,305 (5.2)	8,073 (4.6)
Mpumalanga	85 (6.0)	50 (10.8)	135 (7.2)	4,421 (4.9)	3,744 (4.5)	8,165 (4.7)
North West	99 (7.0)	70 (15.2)	169 (9.0)	5,118 (5.6)	6,991 (8.5)	12,109 (7.0)
Northern Cape	49 (3.5)	13 (2.8)	62 (3.0)	1,930 (2.1)	1,723 (2.1)	3,653 (2.1)
Western Cape	198 (14.0)	32 (6.9)	230 (12.3)	15,194 (16.7)	19,259 (23.3)	34,453 (19.8)
**Number of comorbidities**						
No comorbidities	384 (27.2)	173 (37.5)	557 (29.7)	59,287 (65.0)	14,606 (17.7)	73,893 (42.5)
1 comorbidity	449 (31.8)	167 (36.2)	616 (32.9)	17,498 (19.2)	19,594 (23.7)	37,092 (21.3)
2 comorbidities	351 (24.9)	91 (19.7)	442 (23.6)	8,207 (9.0)	11,967 (14.5)	20,174 (11.6)
≥ 3 comorbidities	227 (16.1)	31(6.7)	258 (13.8)	1,050 (1.2)	5,052 (6.1)	6,102 (3.5)
Unknown	-	-	-	5,189 (5.7)	31,351 (38.0)	36,540 (21.0)
**Required admission to ICU**						
Yes	548 (38.8)	64 (13.9)	612 (32.7)	11,892 (13.0)	2,732 (3.3)	14,624 (8.4)
**Required supplemental Oxygen**						
Yes	1,045 (74.1)	279 (60.4)	1,324 (70.7)	46,733 (51.2)	31,938 (38.7)	78,671 (45.3)
**Required invasive mechanical ventilation**						
Yes	151 (10.7)	28 (6.1)	179 (9.6)	3,487 (3.8)	1,874 (2.3)	5,361 (3.1)

Supplementary Table 5 – UN/Washington Disability Scale Measuring Short Term Disability (Seeing, Hearing, Walking, Remembering, Communication And Self-Care) by 1,873 participants at three months post-discharge from hospital, PCC Study, South Africa

**Condition**	**Better**	**No Change**	**Worse**	**Missing**
Seeing	107 (5.7)	1577 (84.2)	179 (9.6)	10 (0.5)
Hearing	36 (1.9)	1772 (94.6)	53 (2.8)	12 (0.6)
Walking	72 (3.8)	1526 (81.5)	268 (14.3)	7 (0.4)
Remembering	36 (1.9)	1420 (75.8)	409 (21.8)	8 (0.4)
Self-Care	31 (1.7)	1793 (95.7)	40 (2.1)	9 (0.5)
Communication	32 (1.7)	1784 (95.2)	9 (0.5)	48 (2.6)
